# Discussing overweight in children during a regular consultation in general practice: a qualitative study

**DOI:** 10.1186/s12875-020-1088-3

**Published:** 2020-01-28

**Authors:** Joline C. van der Maas, Ronald J. Corbee, Floor M. Kroese, Denise T. D. de Ridder, Rimke C. Vos, Mirjam Nielen, Evelyn Monninkhof

**Affiliations:** 1 Julius Center for Health Sciences and Primary Care, University Medical Center Utrecht, Utrecht University, Utrecht, The Netherlands; 2 Veterinary Medicine, Utrecht University, Utrecht, The Netherlands; 3 Social, Health and Organizational Psychology, Utrecht University, Utrecht, The Netherlands; 4 Department of Public Health and Primary Care / LUMC-Campus The Hague, Leiden University Medical Center, The Hague, The Netherlands

**Keywords:** Overweight, Children, General practice, Regular consultations, Prevention

## Abstract

**Background:**

Overweight in children is a rising problem leading to serious consequences later in life. The Dutch guideline ‘Obesity’ for general practitioners recommends discussing obesity in children regardless of the reason of consultation and provides diagnostic and therapeutic tools. However, limited literature indicates that general practitioners experience barriers to discuss this topic. The aim of this study was to determine current perceived barriers of general practitioners in discussing overweight during a regular consultation in children aged 4 to 12 years and to what extent they discuss the topic. Furthermore, we attempt to get more insight in the specific needs and ideas for improvement among GPs.

**Methods:**

A semi-structured in-depth interview study was conducted. Dutch general practitioners with a broad range of demographic characteristics were invited to participate. The transcripts were analysed using a modified version of the constant comparative method. Using this method, we identified perceived barriers of general practitioners.

**Results:**

Ten general practitioners were included in the study. Four major themes were identified in the interviews: absence of physical or mental complaints related to overweight, internal barriers of the general practitioners, the child’s family background and logistics. Major barriers appeared to be a low consultation rate of these children, the sensitivity of the topic (e.g. fear for children’s or parents’ reactions and/or disturbance of the relation, influence on the self-esteem of the child, resistance in the parents), the absence of a long-standing relation between general practitioner and child or parent, the background of the child and lack of time or prioritizing.

**Conclusion:**

Dutch general practitioners indicate to experience barriers and need tools for how to discuss children’s overweight during regular consultations within the limited time available. The low consultation rate among children aged 4 to 12 years due to lack of physical complaints is mentioned as a new and important barrier. Therefore, the prior focus might be raising awareness among parents concerning overweight in children aged 4 to 12 years and, thereby, stressing the potential supporting role of primary care professionals in tackling the overweight of their child.

## Background

Childhood overweight and obesity is known as a globally rising problem with serious health consequences later in life [1, 2]. In 2016, 12% of the Dutch children aged 4 to 12 years were diagnosed with overweight, and 3% with obesity [3]. Weight status is classified by the Body Mass Index (BMI) and is a measure used to determine childhood overweight and obesity. Overweight is defined as a BMI at or above the 85th percentile and below the 95th percentile for children and teens of the same age and gender. Obesity is defined as a BMI at or above the 95th percentile for children and teens of the same age and gender [4, 5]. Children with obesity have an increased risk for insulin resistance, hypertension and dyslipidaemia, which predisposes them to cardiovascular morbidity in adulthood. In addition, orthopaedic and psychological problems are associated with childhood obesity as well [6, 7].

Primary care professionals are expected to have an important role in the prevention of overweight in The Netherlands [8] as they provide continuity of care [9]. For children aged 0–4 years a designated youth health care physician regularly monitors their health in general, with a limited follow up by the youth health care physician for children aged 4 to 12 years, i.e. only at the age of 5 and 10 [8]. In the past, a general practitioner (GP) would discuss patients’ lifestyle only when symptoms appeared [9]. Nowadays, a more proactive attitude is expected of GPs, especially for children. Some even state it is a GP’s duty to discuss a child’s lifestyle because of the dependent position of the child [10]. However, scarce earlier research in Europe by Paulis et al. (2012) showed that 42% experience barriers in raising the topic of obesity [11]. Although most GPs agreed on having a role in discussing and treating overweight and obesity in children [12, 13], earlier research in 2012 and 2016 showed only 26–38% of GPs discussed the child’s weight during any consultation [11, 12, 14]. GPs appeared reluctant discussing the overweight due to sensitivity of the topic. They feared for a negative influence on the self-esteem of the child and on the relationship with the parents [13, 14]. Therefore, acknowledging the potential consequences of raising the topic regardless of the reason of consultation and tools to raise the topic appropriately is of great importance. Other recognized barriers were lack of motivation and ignorance of parents [12–15], a lack of knowledge and expertise of the GP, and lack of time. International studies also identified these barriers and additionally differences in cultural beliefs and lack of resources as barriers [16–18].

Back in 2010, the ‘Obesity’ guideline from The Dutch College of General Practitioners was introduced [19]. This guideline state that every obese looking child should be examined regardless of the reason of consultation. Whereas the guideline primarily focuses on diagnostics and treatment of overweight and obesity in children, we conducted this study to, get insight in GPs perceived barriers in discussing overweight and obesity during a regular consultation in children aged 4–12 years since the implementation of the guideline in The Netherlands and to what extent they discuss the topic. Furthermore, we attempt to get more insight in the specific needs and ideas for improvement among GPs with a broad range of demographics.

## Methods

### Study design and participants

We used a qualitative design. A purposive sampling strategy was used by approaching GPs with a broad range of demographics to ensure diversity in the study sample (Table 1). GPs were contacted by telephone and email to participate in semi-structured in-depth interviews. Internet, *LinkedIn* and other personal networks were used for recruitment. Demographics as location, name and structure of the general practice, year of graduation and gender of the GP were registered. We continued inviting GPs until we reached topic saturation but aimed to include at least 10 GPs.

**Table 1:** Demographics of the included general practitioners

Number of GP	No. of inhabitants (urbanization rate) (20)	Gender	Graduation year (years’ experience)
#2	*N* = 65.589	Male	2016 (2)
#3	*N* = 9.375	Female	2013 (5)
#4	*N* = 1.775	Female	2007 (11)
#5	*N* = 14.944	Female	1980 (38)
#6	*N* = 41.987	Female	1998 (20)
#7	*N* = 30.030	Male	2013 (5)
#8	*N* = 352.866	Male	2008 (10)
#9	*N* = 176.731	Female	Third year of GP training (0)
#10	*N* = 49.911	Female	2017 (91)
#11	*N* = 41.978	Male	1983 (35)

### Semi-structured in-depth interviews

GPs were interviewed using a pre-specified semi-structured in-depth interview. The main topics of the interviews were based on the barriers mentioned in previous research and were divided into the following subcategories: ‘General information’, ‘Discussing the overweight’, ‘Etiology’, ‘Vision’, ‘Knowledge and expertise’ and ‘Improvement’ (see Additional file 1). The first questions were generic and exploratory. Additional questions were used to further explore barriers addressed by the GP. No adaptations to the interview guide were made during the inclusion period.

### Interview procedure

All in-depth interviews were conducted by one researcher (JM) from November 2017 to January 2018 at a location of the GPs preference (e.g. the general practice or at their home). The interviewer was a Master medical student in her graduation year with interest in general practice. Additionally, she worked during the weekends at the Out of Hours Primary Care Service (‘de Huisartsenpost’). The interviews were recorded with the app ‘Recorder’ using a smartphone and an iBoundary Recording Microphone. For all interviews, a standardized structure was used starting with a short introduction, explaining the purpose of the study and acquiring informed consent for recording the interview. Next, the interview guide with the main questions was used to make sure all participants were interviewed on the same topics uninfluenced by previous experiences of the interviewer (see Appendix). During the interview participants were encouraged to clarify their answer by questions as ‘What ….? ’, ‘Do you have an example?’ or ‘Why....?’. Additional questions were used whenever the participant mentioned the barriers identified in the literature. In order to obtain saturation on a topic, the interviewer asked for amplification using counselling techniques (i.e. offering paraphrase and seeking confirmation that the researcher had understood correctly). Notes were made during the interviews and a member check was performed by giving a summary of the identified barriers followed by confirmation of the GP. The interview ended by showing the researcher’s appreciation for participation. The first interview was a pilot and not included in the analysis. This test interview was used for training purpose of the researcher and to ensure the questions were well understood by the GPs. The interview structure did not need adaptations after the test interview. During the inclusion period, weekly sessions (JM and EM) were scheduled to evaluate the interview progress and to discuss difficulties.

### Data analysis

The audio records were used to transcribe the data verbatim in Microsoft Office Word 2010 during the inclusion period and were imported into QRS NVivo version 11. The interviews were analysed after the inclusion period by using a modified version of the constant comparative method to extract different themes and to develop codes (thematic analysis). Coding was performed by labelling all parts of the interview that were relevant for the research question (JM). Thereafter, sessions of peer debriefing and peer reviewing with one other researcher (EM, clinical epidemiologist) was performed. Inconsistencies were resolved by discussion (EM and JM). After the peer reviewing, a final analysis of the data was performed by evaluating the themes once again. A modified version of the coding outline was developed (JM). Eventually, the coding outline was discussed with all authors.

## Results

In total, interviews with 10 GPs were included in the data analysis. Demographics of the participating GPs, i.e. graduation year, gender, structure and location of the general practice, are presented in Table 1. The duration of the interviews varied from 42 to 71 min. Sixty percent of the participating GPs was female, of whom one in her last year of GP training. Overall, the median of experience in the profession as a GP was 7.5 years. GPs in relatively rural areas with an urbanization rate of *N* < 40.000 had a median of 8 years of experience (*n* = 4), whereas for the relatively urban areas with an urbanization rate of *N* ≥ 40.000 the median of years’ experience was 6 (*n* = 6) [20].

### Absence of physical or mental complaints

The mentioned prevalence of overweight in children visiting the GP in the different general practices varied from a few per week to a few per day. More than half of the GPs stated that the consultation rate of the GP for children aged 4 to 12 years was limited and often not directed at overweight-related (physical or mental) complaints GP #9 mentioned: *‘Well, the problem is … I don’t see children with overweight weekly. It isn’t something we see very frequently... [ …*] *Very young children we see more often and children aged 4 to 12 years we see less. So, it is possible we’re not able to recognize it. (#9)’.*

All GPs diagnosed overweight by visual inspection of the child during a regular consultation. They agreed on having a role in signalling overweight in children, i.e. about half of the GPs indicated a role in guidance or advice and/or in the context of preventive healthcare. On the other hand, almost all GPs argue that the youth health care physician should be responsible in signalling overweight in children during anthropometric follow-up as well. GP #9 mentioned: *‘Initially this is a task for the youth health care physician [during routine contacts]. (#9)’.* Another GP said: *‘I think the system does function. They [the youth health care physician] see the children during routine contacts and they focus on overweight. (#4)’.*

Facilitators mentioned to discuss overweight in children were preventive healthcare, health improvement and personal interest of the GP or focus topic of the general practice for childhood overweight. Most GPs felt more confident discussing the topic if overweight-related complaints were present. During the interviews, several barriers in discussing overweight were identified and discussed according to topic (Table 2).

**Table 2:** Overview of the themes and subthemes identified in the interviews

Themes	Subthemes	Examples	Citations
Absence of physical or mental complaints	Frequency of consultation	e.g. low consultation rate	‘Well, the problem is … I don’t see children with overweight weekly. It isn’t something we see very frequently... […] Very young children we see more often and children aged 4 to 12 years we see less. So, it is possible we’re not able to recognize it. (#9)’
Internal barriers of the GP	Sensitivity of the topic	e.g. fear for patient reaction/ disturbance doctor-patient relation, self-esteem of the child, resistance in the parent	‘It is a very sensitive topic and of course they have heard this before either during bullying or by an aunt saying: ‘you’re getting a little fat’. You know what it’s like with family and their opinions. So, it is already hard for them … that is a barrier to me. I don’t want the child to get hurt. It probably already is? (#6)’
	Motivation of the GP	e.g. negative experience in the past	‘I can imagine when you put a lot of effort in it and it often doesn’t succeed, you might think the next time ‘let it be’. […] And if parents say: ‘we will co-operate’ and you notice change: Yes, then you’re successful. I think it is your experience as well that makes it difficult. Most of the time I think ‘they have to make the effort’ and that is true of course, but apparently, I’m not able to motivate them enough. (#4)’
	Knowledge and skills of the GP		‘This is a sort of grey area for us. (#11)’
	Doctor-patient relation	e.g. absence of doctor-patient relation	‘If you want to discuss it or confront people, it’s important to have a good doctor-patient relation. […] I think it’s the strength of a general practitioner seeing and knowing a patient for a longer time, which makes it easier to anticipate and help to adjust in certain areas. (#8)’
Child’s family background	Child	e.g. culture, socio-economic status	‘Cultural differences might play a role in this. In some cultures, they see overweight as a sign of welfare. For example, it is more common in Antillean or Surinamese children. (#9)’
	Parents	e.g. parents with overweight	‘Yes that [overweight in parents] has definitely an effect. It’s a sensitive topic. Something has been said and done about it a 100.000 times already of course […] Parents often have a wrong diet or are sedentary or have a predisposition for overweight. So, yes that is definitely a barrier. (#6)’
Logistics	Time and prioritizing	e.g. lack of time	‘Due to lack of time, it’s often not discussed […] and lack of time sounds like it’s an external factor, but I think it’s more prioritizing. One prioritizes this in a way that makes it part of the grey area of your time. (#10)’

### Internal barriers of the GP

GPs mentioned several internal barriers during the interviews. The sensitivity of the topic was mentioned by almost all GPs. Mostly, GPs indicated that they were afraid for a negative reaction and/or disturbance of the doctor-patient relationship. GP #9 explained: *‘You are afraid patients will think you meddle too much, leading them not wanting to return [to you as GP], because they think ‘I don’t want to be judged’. But like I said, it is assuming what someone else is thinking. (#9)’*. Furthermore, they consider the self-esteem of the child while discussing the topic. They do not want to blame the child for the overweight or hurt their feelings. GP #6 explained: *‘It is a very sensitive topic and of course they have heard this before either during bullying or by an aunt saying: ‘you’re getting a little fat’. You know what it’s like with family and their opinions. So, it is already hard for them … that is a barrier to me. I don’t want the child to get hurt. It probably already is? (#6)’.* More than half of the GP’s stated potential resistance with the parents as a barrier. Since the overweight was not the reason of consultation, GPs mentioned it as difficult to address the topic. GP #11 said: *‘If it wasn’t the reason for consultation and you’ll try to discuss the child’s overweight, there is often resistance with the mother. She feels attacked at that moment. That she isn’t a good mother. Sometimes mothers have already tried a lot to tackle it, but it appeared not possible [for them] to change the child. (#11)’.*

Half of the GPs stated that they also experienced lack of motivation in discussing the topic since they expect the success rate to be limited, also due to negative experiences in the past. GP #4 illustrated this by saying: *‘I can imagine when you put a lot of effort in it and it often doesn’t succeed, you might think the next time ‘let it be’. [ …*] *And if parents say: ‘we will co-operate’ and you notice change: Yes, then you’re successful. I think it is your experience as well that makes it difficult. Most of the time I think ‘they have to make the effort’ and that is true of course, but apparently, I’m not able to motivate them enough. (#4)’* A facilitator in this was mentioned by one of the GPs, concerning the success rate in discussing previous overweight cases. *‘[ …*] *yes, then you are successful. Then you consider discussing it again [in a next overweight case].’* (#4) Furthermore, one GP mentioned parents can be defensive or downplay the seriousness of the topic. GP #2 exemplified this by saying: *‘In a conversation you can feel if there is an opening. Are people aware and willing to work on it? That is what you sense during a conversation. They can say ‘oh, that’s interesting’ or ‘good point’. I will think about it’ or they can be defensive and downplay it by saying ‘it’s not so bad, doctor’. Then you know it will be hard to motivate them. (#2)’.*

Lack of knowledge and skills, like motivational interviewing and connecting with the patient was mentioned by half of the interviewed GPs as a barrier. GP #4 said about this: *‘Yes, my knowledge is limited. Absolutely. I’m glad there is a nurse practitioner. I think she has more time and experience in motivational interviewing than I have. Let it be her task. That’s fine I think. (#4)’* and GP #6 pointed out *‘It is about understanding and connecting with each other of course. That’s just what changing someone’s behaviour is. [ …*] *Most of the times you do not connect easily [with the patient and/or parents]. (#6)’* Due to a limited knowledge on this topic, one of the GPs suggested to refer to a dietician for example. *‘This is a sort of grey area for us. Yes, then you’ll just refer. Referring a child to a dietician is a possibility, but most of the times the youth health care physician arranges this. (#11)’.*

To discuss overweight, more than half of the GPs mentioned the absence of a long-standing relation between the GP and the child or parent as a barrier. They emphasize the doctor-patient relation can be a facilitator if the GP is familiar with the patient. This makes it easier to know someone’s attitude on the topic, anticipate on a patient’s reaction and to connect with the patient. *‘If you want to discuss it or confront people, it’s important to have a good doctor-patient relation. [ …*] *I think it’s the strength of a general practitioner seeing and knowing a patient for a longer time, which makes it easier to anticipate and help to adjust in certain areas. (#8)’.*

### Child’s family background

Child’s background was mentioned as a barrier by GPs. GP #9 stated: *‘Cultural differences might play a role in this. In some cultures, they see overweight as a sign of welfare. For example, it is more common in Antillean or Surinamese children. (#9)’.* Additionally, parents with overweight are mentioned as a barrier due to the increased sensitivity of the topic in that case as mentioned by GP #6: *‘Yes that [overweight in parents] has definitely an effect. It’s a sensitive topic. Something has been said and done about it a 100.000 times already of course [ …*] *Parents often have a wrong diet or are sedentary or have a predisposition for overweight. So, yes that is definitely a barrier. (#6)’.*

### Logistics

Almost all GPs experienced time as a barrier. Also prioritizing was mentioned by one of the GPs. GP #10 explained: *‘Due to lack of time, it’s often not discussed [ …*] *and lack of time sounds like it’s an external factor, but I think it’s more prioritizing. One prioritizes this in a way that makes it part of the grey area of your time. (#10)’* One GP mentioned a facilitator in this would be project-based screening: […] ‘*when in specific projects, childhood overweight is a focus topic of the general practice and involve other healthcare providers, this GP would discuss overweight more often. (#11)*’.

## Discussion

This qualitative study explored the different challenges of GPs in addressing overweight in children during regular consultations. Despite the introduction of the diagnostic and therapeutic tools provided by the Dutch guideline ‘Obesity’ for GPs in 2010 [19], GPs experience barriers in discussing overweight. We identified four themes of the interview: absence of physical or mental complaints, internal barriers of the GP, the child’s family background and logistics. The major barriers eventually identified were a low consultation rate, the sensitivity of the topic (e.g. fear for patient’s reaction and/or disturbance of the relation, influence on the self-esteem of the child, resistance in the parents), the absence of a long-standing relation between the general practitioner and the child or parent, the background of the child and lack of time or prioritizing. These findings are consistent with previous literature [11–14] and are in this study still identified after the implementation of the guideline back in 2010. Besides, a newly identified barrier concerns the consultation rate of children aged 4 to 12 years. As mentioned before, signalling and providing preventive healthcare might be a task specific for the GP, given the continuity of their care in the general practice. Although GPs in this study agreed on being part of this process, they also emphasize that Dutch children aged 4 to 12 years do not have a high consultation rate due to lack of physical complaints in this age group. In 2015, children aged 4 to 12 years had a consultation rate of about two visits per year [21]. Furthermore, they mostly do not have weight-related complaints which make acknowledging overweight in children challenging in general practice. Therefore, the absence of physical or mental complaints is an important, newly identified, barrier. In international literature, the length of interval between visits of children is also mentioned by health care professionals in general as a barrier [16]. Therefore, the prior focus might be raising awareness among parents concerning overweight in children aged 4 to 12 years and, thereby, stressing the potential supporting role of primary care professionals in tackling the overweight of their child. Despite the introduction of the diagnostic and therapeutic tools provided by the Dutch guideline ‘Obesity’ for GPs in 2010 [19], lack of knowledge and skills was mentioned as a barrier in this study. Paulis et al. (2012) mentioned length and weight are often not measured during non-weight related visits and that GPs do not feel comfortable in treating overweight because of lack of skills or knowledge [11]. GPs stated that the youth health care physician should play an active role as well [12]. However, the continuity of care for these professionals is limited to two consultations in the context of preventive healthcare at the age of 5 and 10 and, therefore, a barrier as well [13]. The barrier of limited routine contact opportunities by preventive healthcare professionals is mentioned in international literature as well [16]. As Schalkwijk et al. (2016) reported, youth health care physicians suggest a yearly check-up as a solution [13]. The sensitivity of the topic overweight appeared as one of the major barriers. This is in line with previous Dutch [11–14] and international research [16, 18, 21–24]. As explained before, the sensitivity lies in the fear of a negative influence on the self-esteem or happiness of the child [13] as well as resistance one may encounter with the parents [11–14, 16, 25]. However, this fear might be unnecessary as uninvited advice might also be appreciated by patients. In a fairly representative sample (*n* = 969) of the Dutch population, 68% agrees on the important role of the GP to give uninvited advice concerning overweight [9]. However, the National Child Measurement Programme (NCMP), a child weight monitoring system in England encountered resistance and concerns from parents about discussing the child’s overweight during regular consultations [25]. This strengthens the thought of raising awareness among parents concerning overweight in children aged 4 to 12 years and the need for tools to raise the topic appropriately. Concerning the barrier of ‘lack of time or prioritizing’ different results have been found in previous studies. While we showed ‘lack of time or prioritizing’ as a barrier in discussing overweight, Paulis et al. (2012) reported that GPs do not experience lack of time as a barrier in raising the subject of ‘overweight’, but do so in the diagnostic work-up [11, 12]. Other studies reported it also as time consuming to discuss [14] or treat overweight [13]. Interestingly, in our study, the doctor-patient relation was mentioned both as barrier and facilitator. GPs are reluctant due to their fear to disturb the relation, which is in line with previous studies [13, 14]. GPs in the present study also referred to the absence of a long-standing relation between the GP and the child or parent as a barrier, and therefore, the lack of knowledge of the patients’ social and medical situation. This might be explained by the inclusion of acting GPs in this study implicating the absence of a longstanding doctor-patient relation. Investment in a firm relationship accompanied by more knowledge as mentioned before might facilitate discussing overweight. Barriers that were previously mentioned, but not identified in this study are, for example, lack of effective interventions and lack of resources and reimbursement [18, 22–24, 26]. This might be explained by the organization of the health care systems in different countries [27, 28]. For example, rural areas are more common in certain regions of Western countries such as the United States of America (USA), Canada or Australia, where lack of resources is more likely to be a barrier. The access to overweight related projects and/or healthcare providers might be limited therefore. However, Dutch GPs mention a lack of (effective) overweight related projects as well [14]. Furthermore, nurse practitioners have an important role in preventive health care in the USA. They provide general health visits or yearly check-ups and may have more specialized knowledge and skills on addressing these sensitive issues than Dutch GPs have [27]. Therefore, the applicability of these results is limited to the Dutch population.

### Limitations and strengths

Research concerning barriers in discussing overweight in children is limited in The Netherlands. This study provided more insight in GPs’ perceptions. Through data collection using semi-structured in-depth interviews, wide insight into the barriers experienced by GPs in discussing overweight in children during regular consultations was provided. Furthermore, we included general practices with a broad range of demographics representing patients of different social economic status. This study was limited by several factors. The research area covered only two provinces in The Netherlands. Thereby, we might have missed other cultural areas with different ethnic groups. Inclusion of GPs may have been affected by the interest of the GP (e.g. participating GPs may be more interested in the topic overweight). One of the approached GPs for example refused to participate due to a different interest area of the general practice. Another limitation concerns the design of interviewing and coding. Coding was not independently performed by two researchers. However, a second researcher (EM, clinical epidemiologist) was closely involved in the coding process, i.e. in discussing the codes during the analyses and developing the final coding outline. There was no GP involved in the data analysis. However, the interviewer, was a graduating Master medical student with interest in general practice. Additionally, the interviewer worked during the weekends at the Out of Hours Primary Care Service (‘de Huisartsenpost’). Finally, In future research, a mixed-method design, including quantitative research, would be recommendable to confirm the results and conclusions [29].

## Conclusion

In conclusion, Dutch general practitioners seem to experience barriers in discussing overweight during regular consultations despite the availability of the diagnostic and therapeutic tools in the Dutch guideline ‘Obesity’ for general practitioners introduced in 2010 [19]. The limited consultation rate in children aged 4 to 12 years due to lack of physical complaints is one of the newly and most important barriers hindering the signalling role of the GP. Other newly identified barriers were a patient’s background (e.g. culture) and the absence of a long-standing relation between the GP and the child or parent. Important barriers as the sensitivity of the issue and lack of time or prioritizing were identified once again. This study implicates room for improvement in discussing overweight during regular consultations. Improvement of GPs’ knowledge and/or skills and awareness in parents may lead to decreasing the sensitivity of the topic. However, due to absence of physical complaints in children aged 4 to 12 years, the prior focus might be raising awareness among parents concerning overweight in children aged 4 to 12 years and, thereby, stressing the potential supporting role of primary care professionals in tackling the overweight of their child.

## Supplementary information

**Additional file 1: Questionaire.** Semi-structured in-depth interview

## Data Availability

The datasets used during the current study are available from the corresponding author on reasonable request.

## Abbreviations

BMI: Body Mass Index
EM: Evelyn Monninkhof
GP: General practitioner
JM: Joline van der Maas
NCMP: National Child Measurement Programme
