# Supplementary material for: Discussing overweight in children during a regular consultation in general practice: a qualitative study
Source: BMC Fam Pract. 2020 Jan 28;21:18. doi: 10.1186/s12875-020-1088-3 (PMC6986030; doi:10.1186/s12875-020-1088-3)
Supplement: Supplementary file 1 — Additional file 1: Questionaire. Semi-structured in-depth interview [file 12875_2020_1088_MOESM1_ESM.docx]

**Appendix 1. Semi-structured in-depth interview**

| **Personal data** |
| --- |

General practice: Date: Time:

Name general practitioner:

Location general practice: Phone number:

Practice type:

Year of graduation:

| **Key questions** |
| --- |

General information

1. When is a child overweight according to you?
2. What percentage of the children you encounter during a working week has overweight in your general practice?

Discussing overweight

1. When do you discuss the overweight with a child and/or parent?
2. How do you discuss overweight during a non-overweight related consult?
3. What kind of problems do you experience while discussing overweight?

Etiology

1. What is the most important cause of overweight according to you?
2. In what extent are parents responsible for a child’s overweight?
3. In what extent is a child influenced by socioeconomic class?

Vision

1. What is the task of the general practitioner regarding overweight in children according to you?
2. What is your personal motivator to discuss overweight?
3. Can you tell me about the allocation of duties within the general practice regarding overweight in children?
4. Can you tell me about the vision within your general practice regarding overweight in children?

Knowledge and skills

1. What treatment strategy do you apply after diagnosing the overweight in children?
2. What do you think about the information available for general practitioners regarding overweight in children?
3. What do you think about the information available for children and parents regarding overweight and how to tackle this?
4. Do you feel the need to follow any training regarding overweight in children? And if yes, on what aspect(s)?

Improvement

1. Are there tips and tricks you apply while discussing overweight? And if yes, which one?
2. What do you think should change in order to discuss overweight more often during non-overweight related consultations?
